# Normalizing for individual cell population context in the analysis of high-content cellular screens

**DOI:** 10.1186/1471-2105-12-485

**Published:** 2011-12-20

**Authors:** Bettina Knapp, Ilka Rebhan, Anil Kumar, Petr Matula, Narsis A Kiani, Marco Binder, Holger Erfle, Karl Rohr, Roland Eils, Ralf Bartenschlager, Lars Kaderali

**Affiliations:** 1 Heidelberg University, ViroQuant Research Group Modeling, BioQuant BQ26, Im Neuenheimer Feld 267, 69120 Heidelberg, Germany; 2 Heidelberg University, Department of Infectious Diseases, Molecular Virology, Im Neuenheimer Feld 345, 69120 Heidelberg, Germany; 3 Heidelberg University, Integrative Bioinformatics and Systems Biology, BioQuant, Im Neuenheimer Feld 267, 69120 Heidelberg, Germany; 4 Center for Biomedical Image Analysis, Faculty of Informatics, Masaryk University, 602 00 Brno, Czech Republic; 5 Heidelberg University, BioQuant/Cellnetworks RNAi Screening Facility, Im Neuenheimer Feld 267, 69120 Heidelberg, Germany; 6 University of Technology Dresden, Medical Faculty, Institute for Medical Informatics and Biometry, Fetscherstrasse 74, 01307 Dresden, Germany

## Abstract

**Background:**

High-content, high-throughput RNA interference (RNAi) offers unprecedented possibilities to elucidate gene function and involvement in biological processes. Microscopy based screening allows phenotypic observations at the level of individual cells. It was recently shown that a cell's population context significantly influences results. However, standard analysis methods for cellular screens do not currently take individual cell data into account unless this is important for the phenotype of interest, i.e. when studying cell morphology.

**Results:**

We present a method that normalizes and statistically scores microscopy based RNAi screens, exploiting individual cell information of hundreds of cells per knockdown. Each cell's individual population context is employed in normalization. We present results on two infection screens for hepatitis C and dengue virus, both showing considerable effects on observed phenotypes due to population context. In addition, we show on a non-virus screen that these effects can be found also in RNAi data in the absence of any virus. Using our approach to normalize against these effects we achieve improved performance in comparison to an analysis without this normalization and hit scoring strategy. Furthermore, our approach results in the identification of considerably more significantly enriched pathways in hepatitis C virus replication than using a standard analysis approach.

**Conclusions:**

Using a cell-based analysis and normalization for population context, we achieve improved sensitivity and specificity not only on a individual protein level, but especially also on a pathway level. This leads to the identification of new host dependency factors of the hepatitis C and dengue viruses and higher reproducibility of results.

## Background

RNAi screening has emerged as a novel technique to functionally characterize genes in living cells. Using short interfering RNAs (siRNAs), the technique allows sequence-specific gene silencing in a high-throughput fashion. This has successfully been used in several large-scale screens, for example, focusing on genes involved in mitosis [1], immune response [2] or viral infection [3,4]. The platform can be combined with automated microscopy, which then allows the acquisition of multi-parametric phenotypes of hundreds of cells per knockdown in a high-throughput fashion, yielding large data sets and unprecedented opportunities for functional genomics [5,6]. Unlike siRNA screens using bulk measurements, microscopy-based screening offers a much more detailed view, since for each siRNA single-cell observations are available. This increased information can be exploited to identify genes that cause different morphological phenotypes, for example genes that are related to focal adhesion or the cell cycle [1,7-9], but offers also new possibilities to normalize data at the level of individual cells.

Whilst a large variety of different normalization methods exist for microarrays, only some standard techniques have been adapted so far for RNAi data [10-12]. Even for microscopy-based screens, most studies calculate the mean or median fluorescence intensity of all cells in the same well, and use these summarized values to normalize within and between different experiments [11,13-16]. Although the loess normalization is commonly used [11,13,17] to normalize the average cell signal intensities against the number of cells within one spot, extensive normalization based on individual cell data has not been done so far. Depending on the experimental setup, this results in a loss of information of hundreds of individual cell measurements, with associated detrimental effect on statistical power. Furthermore, population context of individual cells is completely disregarded, which is in strong contrast to a recent study performed by Snijder et al. [18]. These authors show that a cell's population context has considerable influence on endocytosis and viral infection. Although their work studies population effects in the absence of siRNA knockdowns, the results strongly advocate the use of high-content microscopy and appropriate cell-based data analysis methods for RNAi screens.

Only a few methods so far have used individual cell information from high-content microscopy-based screens, and to our knowledge none to date have used the population context for data normalization. Fuchs and co-authors recently proposed the use of multiparametric phenotypic profiles of RNAi screening data to cluster genes and to discover novel gene functions [8]. Their prediction is mainly based on morphological changes of individual cells within a cell population. Suratanee et al. have proposed the use of a spatial clustering approach to identify siRNA knockdowns involved in viral infection [19]. Their approach is based on the assumption that viruses mainly spread by cell-to-cell contacts. The authors assume that infected cells form clusters in microscopy images, and use Ripley's k function to identify knockdowns resulting in a disturbed clustering of cells.

In this paper, we present a novel method to analyze high-throughput, high-content cellular assays, based on single cell measurements. We show results on two viral screens of hepatitis C virus (HCV) and dengue virus (DENV). We observed considerable effects of cell population context on infection, and thus normalize the measurements of each individual cell against its population context. We furthermore implement within-plate and between-plate normalization methods for microscopy screening data. We then identify statistically significant knockdowns by taking fluorescence signal measurements of all individual cells in the screen. Using this approach, we were able to reconfirm several known and identify new host dependency factors for both HCV and DENV. The methods are implemented in the statistical programming language R http://www.r-project.org, and are available from the journals website (Additional file 1).

## Results and Discussion

We conducted two different high-content RNAi primary screens targeting the same set of 719 human kinases in a virus infection setting. The first screen aimed to identify host cell factors involved in HCV infection, the second screen focused on DENV. Both screens were carried out on cell arrays [20]. Results of the HCV screen have previously been published using an analysis based on average intensities per spot [17], and the screen has consequently been re-analyzed using a clustering approach on the raw microscopy data [19]. Using this screen then, a comparison can be made between these previous approaches and the method proposed here. The DENV screen has not been published before.

### Cell-to-cell variability in RNA interference screens

**Figure 1:**
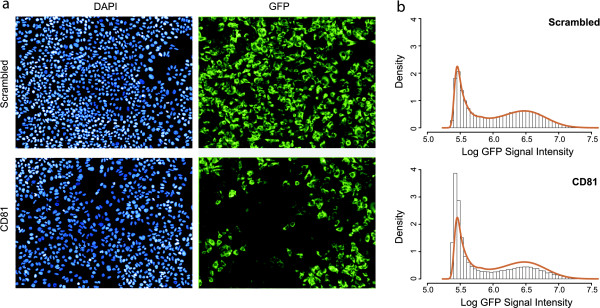
**Signal intensity measurements of controls**. **(a) **Microscopy images for negative (scrambled) and positive controls (*CD81*) in the hepatitis C virus infection screen. The left panel shows the nuclei (DAPI) channel, the right panel the virus signal (GFP). A clear effect of the *CD81 *knockdown showing significant reduction of the GFP signal is visible, while cell counts are not affected. **(b) **Histogram of single cell log-transformed GFP intensity values of the positive and negative controls in the HCV screen, measuring hepatitis C virus infection efficiency. The brown curve indicates the signal distribution of all cells in the entire screen, perfectly matching the histogram of the negative, but not of the positive controls. Notably, two populations of cells are discernible both in the positive and negative controls, corresponding to infected and non-infected cells. Positive and negative controls differ in the sizes of these subpopulations, and thus the shape of the overall distribution of GFP intensity values.

We observed considerable cell-to-cell variability in both screens, even within the same spot. Figure 1a shows microscopy images for a negative control (scrambled siRNA) and the biological positive control *CD81*, in the HCV screen. Two color channels are shown for the same spot: Cell nuclei were stained using DAPI (left images) and a GFP readout was used for viral infection of the same cells (right images). Variability between individual cells is shown in Figure 1b, which plots the distribution of log-GFP signal intensity values of the single cell measurements of negative (top) and positive (bottom) controls. Of note, in both cases two populations corresponding to infected and non-infected cells can be observed, but the positive and negative controls differ in the distribution of cells between the two groups. The solid brown line shows the log-GFP intensity distribution of all cells in the entire screen (without controls). Importantly, the negative controls exactly fit the shape of the curve, whereas *CD81 *does not - indicating that the full screen can be used as a quasi-negative control, and that most knockdowns in the screen have no effect on viral infection. The number of cells in wells with positive versus negative controls is roughly the same (HCV: 326.82 ± 5.44 vs. 338.32 ± 3.43 mean ± std. error, p = 0.11 using two-sided, two-sample Welch's t-test; DENV: 279.81 ± 7.64 vs. 287.0 ± 5.3; p = 0.46), indicating no significant apoptotic effect of the corresponding siRNA knockdowns.

To further characterize cell-to-cell variability, we fitted a Gaussian mixture model to normalized log-transformed intensity values. This fit shows a clear bimodal distribution of the data with approximately normally distributed components (μ = 0.34 ± 1.25*10^-4 ^non-infected and μ = -0.5 ± 3.63*10^-5 ^infected cells for HCV; μ = 0.21 ± 1.71*10^-4 ^non-infected and μ = -0.72 ± 1.71*10^-4 ^infected cells for DENV). Estimation of the mixture coefficients for the positive and negative controls confirm that there are clear differences. For the positive controls of HCV (DENV) there is an uninfected cell component probability of p_u_^+ ^= 0.61 ± 0.002 (p_u_^+ ^= 0.42 ± 0.002), and for the negative controls p_u_^- ^= 0.4 ± 0.001 (p_u_^- ^= 0.21 ± 0.001). This confirms that siRNA knockdowns of genes required for HCV and DENV infection or replication (dependency factors) have a higher proportion of cells with weak signal intensities. Clearly, under optimal conditions (perfect transfection, knockdown and infection efficiencies), only background GFP intensity for positive and maximal intensity for negative controls would be expected.

### Population context influences infection

We next studied the effect of population parameters on hepatitis C virus and Dengue virus infection, using a procedure similar to the one proposed by Snijder et al. [18]. Notably, while the analysis done by these authors considers the effect of population context on viral infection in the absence of any RNAi perturbation, we here present for the first time results including knockdowns. Furthermore, results shown here are based on chambered coverglass slides (LabTeks), whereas Snijder et al. used 96 well plates, with considerable differences in cell numbers and cell densities between these two platforms.

We computed six different population features for each cell based on the DAPI and GFP stains: (1) Size of the cell, (2) number of cells in the same spot, (3) location of the cell in a local population (center or edge of a local cell population), (4) local cell density and (5) shape of the cell nucleus (1/circularity of the cell nucleus). We note that since no stain for the cell cytoplasm is available, nuclear size is used as an approximation to cell size, as previously proposed by Snijder et al. We furthermore calculated four technical features: (1) location of the cell in the spot (center of spot or at the border), (2) row effects of cell location (median signal intensity of all cells in corresponding row on LabTek), (3) column effect of cell location (median signal intensity of all cells in corresponding column on LabTek), and (4) plate effect (median signal intensity of all cells on corresponding LabTek).

Due to the large number of individual cells, we grouped cells with similar properties into 20 discrete bins per feature. For each of the 5 population context and 4 technical features, we then computed the average within-bin and between-bin standard deviation of viral infection. The ratio of these two values provides a direct measure to assess the influences of the corresponding population or technical parameter on viral infection. A similar procedure has been used by Snijder et al to compute the population effects.

**Figure 2:**
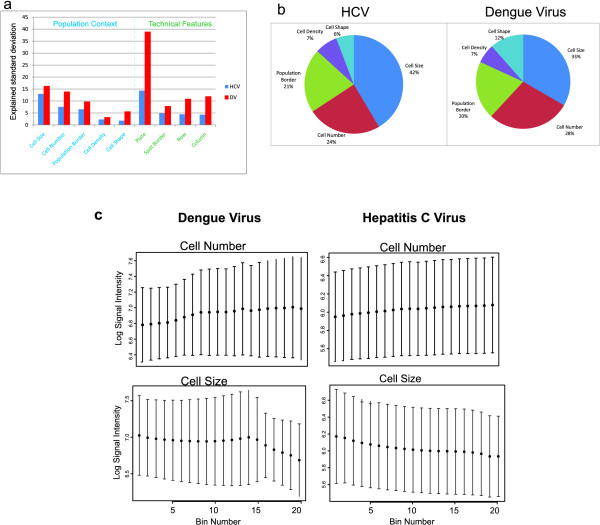
**Results of the population context and technical feature computations**. **(a) **Explained standard deviation of cell population context and technical features with respect to single-cell viral infection efficiency. Shown are the ratios of the between-bin standard deviation to the average within-bin standard deviation, as a measure of the fraction of the variation explained by the cell population or technical feature under consideration. Blue: Hepatitis C virus (HCV) screen, red: Dengue virus (DENV) screen. While overall variability is larger for DENV, the relative effect of individual features is comparable between both viruses under consideration. Importantly, a clear effect of cell population context on viral infection is discernible. **(b) **Percentage of explained standard deviation for cell context features for HCV and DENV, relative to the total variability due to cell population context. **(c) **Mean and standard deviation of the two population context features which are most important, namely Cell Size and Cell number of the two screens in the twenty individual bins.

Figure 2a shows the explained standard deviation of viral infection for each of the five population and four technical features we computed. The greatest influence on signal intensity both in HCV and in DENV was due to plate effects, with 14.35% (HCV) and 38.9% (DENV) of the standard deviation explained by this parameter. This was followed by cell size (12.96% vs. 16.24% explained variation) as the second most important parameter in both screens. Spatial effects on the LabTeks amounted to between 4.29% to 11.9% of the total variation. Individual population features explained between 1.8% to 16.24% of the total variation. We note that the overall variance was higher in the DENV screen (coefficient of variation of raw signal intensities CV = 0.595 DENV, in comparison to CV = 0.546 HCV).

Figure 2b shows the relative importance of the five population context parameters on HCV and DENV infection. In both viruses, cell size dominates with 42% (HCV) and 33% (DENV) of the variance due to population context. Cell number (24% HCV, 28% DENV) and cell position in local population (21% HCV, 20% DENV) are the second and third most important parameters. Local cell density (7% in both viruses) and cell shape (6% HCV, 12% DENV) both still play an important role, but relatively minor when compared to other factors. This is in contrast to the previous findings of Snijder and co-authors, who reported that the location of a cell at the edge or in the middle of a cell cluster is the main contributing factor for dengue virus infection [18]. This difference may be due to different conditions in 96 well plates versus on chambered coverglass slides (LabTeks), and probably also due to different cell lines used in the two experiments (Huh7.5 vs. HeLa).

### Single cell based normalization and hit selection

Figure 2c gives the mean and standard deviation for each of the twenty bins of the two most important population context features on both screens (for the other two, non-binary, population context features see Additional file 2). This shows, for example, that in the DENV and HCV screen, the log signal virus intensity is smaller the larger the cells. In contrast, the signal intensity is higher the greater the cell number. To test whether the effects of the individual features on the virus signal intensities are linear, we used a Harvey-Collier test for linearity computed on the log signal intensities and the raw features (without binning). The results show that all features are significantly nonlinear (p-values ≤ 2.2*10^-16 ^for all features of the DENV and HCV screen, except for the Spot border feature of HCV with a p-value ≤ 2.987*10^-7 ^and the Column feature of DENV with a p-value ≤ 1.22*10^-4^).

Due to these nonlinear effects of the population context and technical features, we used multivariate adaptive regression splines (MARS) on the full data (without binning) to estimate the influence of the features on HCV and DENV infection [21]. The fitted model was then used to correct raw intensity values, by subtracting estimated effects from individual cell measurements.

**Figure 3:**
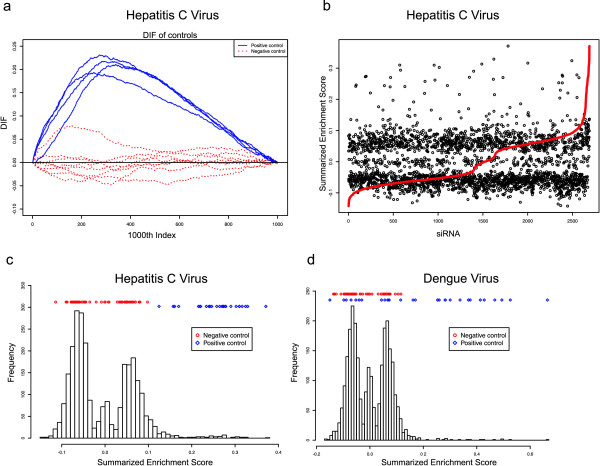
**Results of the *ES *compuations**. **(a) **Computed DIF values for the positive (blue) and negative (red) controls of a randomly selected plate from the hepatitis C virus screen. Note that four positive and seven negative controls were spotted on this plate. DIF measures the difference between the running sums computed for each siRNA or well in the screen, describing the enrichment of cells from the corresponding well towards high or low signal intensities. The enrichment score ES is the maximum DIF value observed for a particular spot/siRNA under consideration. **(b) **Summarized enrichment scores ES of each siRNA in the HCV screen. Replicate enrichment scores were summarized using the median, siRNAs were plotted in the sequence as spotted on the plates. The red line shows the siRNAs sorted by increasing ES. **(c, d) **Distribution of the summarized enrichment scores ES of the full HCV and DENV screens, summarizing replicates using the median. The location of positive and negative control scores is indicated by red circles for the negative and by blue diamonds for the positive controls.

We next developed a statistical test to identify siRNA knockdowns showing a significant effect on viral replication, borrowing ideas from gene set enrichment analysis (GSEA) as proposed by Sweet-Cordero et al. [22,23]. This procedure is essentially a Kolmogorov-Smirnov test on running sums over ranked intensity values, see methods [24]. We are looking for siRNAs that lead to a shift in the distribution of individual cell signal intensities away from the distribution of negative controls, towards an increased number of non-infected cells showing only background fluorescence. The basic principle employed in this procedure is to sort all cells in the entire screen according to their measured viral signal intensity. Two running sums $R{S_{G}}_{{}_{k}}$ and $RS_{{Ḡ}_{k}}$ are then computed, counting the fraction of cells treated or not treated with a particular siRNA *G_k _*of interest as the signal intensity is gradually increased (see Equation 1 and Equation 2, methods). The difference *DIF *between these two values measures the enrichment of cells with respective intensity values in the knockdown of interest (Equation 3). Figure 3a shows *DIF *values of the positive and negative controls over the indices of the sorted cell intensity values of a randomly selected plate of the HCV screen, illustrating the clear differences observable between positive and negative controls. Whereas the *DIF *value for the positive controls is increasing until a maximum of about 0.2 is reached and then decreases again, the *DIF *value of the negative controls is fluctuating around zero. The *enrichment score ES *is defined as the maximum deviation of *DIF *from zero. Figure 3b shows the median enrichment score *ES *for each siRNA in the whole screen. The red curve illustrates the *ES *sorted by increasing order.

The Figures 3c and 3d show the distribution of the enrichment scores *ES *of all siRNAs in the HCV and DENV screens. Scores of the positive controls are indicated by blue diamonds, and for the negative controls by red circles at the top of the plot. Interestingly, while the positive and negative controls are perfectly separated for the HCV data, some of the positive controls in the DENV screen are not working properly. Since other quality indicators of the affected plates in the DENV screen were fine (correlation between replicates, other controls on the same plates, statistics of plate and location effects) but we generally observed higher variability in the DENV screen, we decided not to remove the full plate for the affected controls.

An interesting observation is the three peaks of the distribution in Figures 3c and 3d, which seems counter-intuitive. This tri-modality comes from summarizing the replicates using the *median *of the *ES *of the replicate measurements. Since there is an even number of replicates (twelve for HCV and six for DENV) the mean of the two central elements is used as median, and gives a value around zero, if exactly one half of the replicates has positive and the other half has negative *ES *values. The siRNAs that have the majority of replicates with positive or negative *ES *value occur in the right or left peak, respectively. This tri-modality effect is thus an artifact of summarizing an even number of replicates using the median, and does not occur when taking the mean - which however is less robust to outlier siRNAs.

The *ES *were then subjected to a nonparametric statistical test to identify gene knockdowns that significantly decrease viral replication, using a significance level of adjusted p-values of *α *= 0.05 and an *ES *larger than 1.5 times the standard deviation of the median over the replicates for the calculated *ES *of each LabTek.

### Hepatitis C virus host dependency factors

Using the normalization for cell population context and subsequent hit scoring as described above resulted in a list of 54 host dependency factors significantly reducing HCV replication (see Additional file 3). We compared identified hits to results obtained using a statistical analysis based on averaged intensity values per well (AVERAGE), as previously published [17]. A z-score threshold of 1.5 and a significance level of 5% were used for hit identification in the AVERAGE method. We furthermore compared results with the analysis method based on Ripley's k function recently proposed by Suratanee and coauthors [19], with significance level 5% and negative clustering scores (RIPLEY).

**Figure 4:**
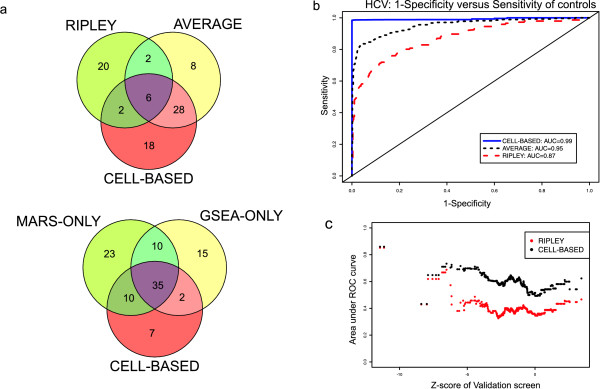
**Results of the different analysis methods on the HCV data**. **(a) Upper plot: **Venn-Diagram of the hits at the gene level using the CELL-BASED, AVERAGE and RIPLEY analysis methods. A total of 30 host dependency factors were identified using RIPLEY, 44 host dependency factors were identified using the AVERAGE, and 88 factors were identified using the CELL-BASED approach. 39 genes were commonly identified between CELL-BASED and AVERAGE, but only 8 genes common between AVERAGE and RIPLEY and only 10 common genes between RIPLEY and CELL-BASED. **Lower plot: **Venn-Diagram of the hits at the gene level using the CELL-BASED, GSEA-ONLY and MARS-ONLY analysis methods **(b) **Receiver operator characteristic (ROC) analysis of correct identification of positive and negative controls in the HCV screen. Sensitivity and specificity of the recognition of positive and negative controls was computed for different thresholds on computed scores or significance levels, using the AVERAGE, CELL-BASED and RIPLEY approaches. Hit thresholds were computed on z-score, clustering scores and ES, respectively. ROC curves were generated by varying these thresholds, and plotting sensitivity over 1-specificity. Pink: CELL-BASED, Black: AVERAGE, Red: RIPLEY. The area under each curve was computed to obtain a single value measuring the quality of the control classification. AUC values of 0.5 correspond to random guessing, AUC values of 1 to perfect classification. Achieved AUC values varied between 0.87 for RIPLEY, 0.95 for AVERAGE, and 0.99 for CELL-BASED, showing best performance of the CELL-BASED approach. **(c) **Comparison of obtained area under the ROC curve (AUC) values on an HCV validation screen, using hit genes identified in the primary screen using the CELL-BASED and RIPLEY methods. In brief, hit genes identified using the AVERAGE method on the primary screen were subjected to a secondary validation screen. The intersection of predicted hits on the primary screen using the RIPLEY and CELL-BASED approaches with genes screened in the validation screen was used to compute ROC curves and AUC values for CELL-BASED and RIPLEY. Shown are AUC-values over different z-score thresholds on the validation screen.

Surprisingly, there are considerable differences in identified hits, with only 6 gene knockdowns overlapping between all three methods, compare Figure 4a (upper VENN-Diagram). Genes in the overlap consistently identified by all approaches are the positive controls *HCV321 *and *HCV138 *directly targeting the viral RNA genome, and *CD81*, the main entry receptor required by HCV. The remaining three overlapping hits are phosphatidylinositol 4-kinase alpha (*PI4KA*) [4,25-29], casein kinase II subunit alpha (*CSNK2A1*) [30], which are known to be related to the HCV replication cycle and fms-related tyrosine kinase 4 (*FLT4*) which has been suggested to play a role in HCV in earlier publications [19,31]. Out of the 44 genes identified using the AVERAGE approach, 34 genes were also found using the cell-based method presented here (CELL-BASED), accounting for an overlap of 77.3% and indicating high agreement between CELL-BASED and AVERAGE results. On the other hand, only 10 of the 30 genes identified using the RIPLEY approach could also be confirmed with the other methods (33.3%), and only 8 out of 44 genes identified using AVERAGE were also found by RIPLEY (18.2%).

Most dependency factors were identified using the CELL-BASED approach (54) (AVERAGE: 44 and RIPLEY: 30). While we expect higher statistical power when using individual cell data, the question arises how reliable and reproducible identified hits are. We therefore first evaluated how reliably the positive and negative controls were identified with the three approaches, by computing sensitivity and specificity and analyzing receiver operator characteristic (ROC) curves for all three analysis methods based on the individual control spots on each plate. ROC plots show tradeoffs between sensitivity and specificity for different thresholds on scores or p-values used for hit-calling. Random guessing would correspond to a diagonal line in the ROC plot, whereas a perfect predictor would yield sensitivity and specificity values of one. ROC curves can be summarized further by computing the area under the curve (AUC), which is a value between 0.5 (random guessing) and 1 (perfect classification). Figure 4b shows ROC curves for AVERAGE, RIPLEY and CELL-BASED. Although all ROC curves are significantly better than random guessing, AUC values show that CELL-BASED outperforms the other two approaches in scoring controls almost perfectly (CELL-BASED: 0.989, AVERAGE: 0.95, RIPLEY: 0.87). Results are superior in recognizing positive and negative controls in terms of both sensitivity and specificity of. For the AVERAGE method a loess normalization was used to normalize for general trends between the mean viral signal intensities and the number of cells within one spot. Furthermore, b-score normalization was used to normalize against spatial plate effects. The AUC values show, that the AVERAGE method cannot minimize the introduction of false positive and false negative controls on a single spot level as well as the CELL-BASED method.

Since our analysis approach consists of two independent methods (normalization against the population context features using MARS and the statistical test based on the idea of GSEA) we assessed which of the two methods contributed the most to the increased performance of classifying the controls. To do this, we used each of the two methods independently on the HCV data. For the first method (MARS-ONLY) we took raw log virus signal intensity values and normalized them against the features. Then, we used RNAither [11] to summarize the cell intensities of one spot using their mean and computed z-scores for each spot. By applying a threshold of 1.5 times the standard deviation of the z-scores of each replicate we defined hits on an individual spot level. This procedure thus exploits the single cell information for normalization, but nor for hit-calling. We note, that the calculation of significance levels for each spot is not possible in this method. For the second method (GSEA-ONLY) we calculated *ES *on the raw log virus signal intensities and used the nonparametric statistical test based on permutations to calculate p-values for each spot - exploiting the full information of the individual cells in the statistical test. We used Bonferroni corrected p-values ≤ 0.05 and *ES*≥1.5 times the standard deviation of median summarized replicate *ES *for finding individual spots which significantly decrease viral replication. We performed for both methods a ROC analysis on individual control spots and computed AUC values. MARS-ONLY results in an AUC of 0.971 and GSEA-ONLY in an AUC value of 0.987.

In summary, both individual methods are able to yield improved results in comparison to the AVERAGE (AUC = 0.95) and RIPLEY (AUC = 0.87) methods. The combination of MARS-ONLY and GSEA-ONLY in our CELL-BASED approach gives the best result when compared to the stand alone methods.

In addition, we summarized replicate measurements of MARS-ONLY by taking their median and used a two-sample, two-sided Welch's t-test to define significance values for the individual siRNAs. We used an alpha threshold of 0.05 on uncorrected p-values and 1.5 times the standard deviation of z-scores to define significant siRNAs. The same was done for GSEA-ONLY, although p-values for individual siRNAs have been calculated based on the *ES *using the nonparametric test. Of the resulting hits for GSEA-ONLY, MARS-ONLY and CELL-BASED there are overlapping genes 35 (see Figure 4a lower VENN-Diagram and S1). Among the 54 hits found using the combined CELL-BASED method 47 (87%) were also found with the two independent methods.

Genes previously identified by Reiss et al. using the AVERAGE method were tested in a secondary validation screen by the same authors. We used this screen to further assess our method. Using the subset of genes tested in the validation screen, we computed sensitivity and specificity for varying z-score thresholds for the validation screen, varying *ES *score thresholds and adjusted p-values ≤ 0.05 for selecting hits of the CELL-BASED method and varying p-values and negative clustering scores for the RIPLEY method. It can be noted that an evaluation of the AVERAGE method with this data is not possible, since this approach was used to select genes for the validation screen, and hence true and false negatives cannot be computed for AVERAGE (negatives were not included in the validation screen). Figure 4c shows the AUC values over different increasing z-score thresholds on the validation screen, for CELL-BASED and RIPLEY. Again, CELL-BASED shows superior sensitivity and specificity (results of GSEA-ONLY and MARS-ONLY look similar to CELL-BASED and are thus not shown).

### Pathway analysis and functional annotation of host dependency factors

We identified 54 host dependency factors (HDF) for HCV and 57 HDFs for DENV, see Additional files 3 and 4. These were further mapped to KEGG and Biocarta, and functional enrichment tests were carried out to select significantly enriched processes using DAVID http://david.abcc.ncifcrf.gov/, see Additional files 5 and 6. We identified 20 pathways with a p-value smaller than 0.05 to be significantly involved in HCV using the hits identified with our CELL-BASED approach. This is ten times the number of pathways which are identified to be significantly enriched using the hits of the AVERAGE method. In this method, only two pathways were found, namely Purine metabolism, which was also identified with the CELL-BASED method, and Axon guidance. Using the hit list of the RIPLEY approach no pathways could be identified as significantly enriched. This clearly shows, that our approach results not only an increased sensitivity and specificity on an individual siRNA level, but also an increased sensitivity on the pathway level.

Enriched pathways for HCV include endocytosis, focal adhesion, signaling in the immune system, regulation of the actin cytoskeleton, and the *ErbB *and *MAP *kinase signaling pathways. All of these pathways have previously been reported by Reiss and colleagues [17] by pooling their screen with other previously published screens. Notably, with the approach presented here, we identified the same pathways without the additional information of other screens, again indicating higher sensitivity of the CELL-BASED approach. Several additional pathways were also identified, including purine metabolism, *TLR *signaling and several cancer-associated pathways.

Additional file 6 shows enriched pathways of the DENV screen. Although the overlap between HCV and DENV HDFs at the gene level is only seven genes, significant overlap can be observed at the level of pathways. Enriched pathways again include focal adhesion, immune signaling, and the *ErbB *and *MAP *kinase pathways, reflecting the close evolutionary relationship of the two viruses.

### Population context of non-virus screens

Apart from virus infection, the impact of many other- if not all- "bioactive" treatments on cells most likely depends on the properties of the individual cells and their context. A pristine and ubiquitous example is the transfection of cells using liposomal reagents. Every wet lab biologist will know intuitively, that efficiency of transfection strongly depends on the confluency of the culture. While this is true and relevant for bulk measurements of larger formats, it becomes critical when dealing with the low cell counts typically found in the wells or spots of high-throughput screening formats. In an imaging-based screen of an innate immune signaling pathway, which shall be published elsewhere, cells were again reverse transfected with siRNA on spots of LabTek chamber slides. To determine the impact of gene silencing on the pathway under investigation, signaling was triggered by transfection of the cells with a defined stimulus and a few hours later, pathway activation in individual cells was assessed by microscopy of a fluorescent reporter. Across the whole screen (ca. 2.4 Mio. cells were analyzed), we could detect a strict correlation between population context and the rate of pathway activation, due to the vastly different susceptibility for liposomal transfection among cells growing in different micro-contexts. To quantify this we calculated the explained standard deviation of the rate of pathway activation by the population context features for each plate. The mean and standard deviation across replicated plates of the individual population features are for Cell Size: 8.1 ± 2.57, Cell Density: 9.24 ± 3.5, Cell Number: 8.8 ± 2.4, Cell Shape: 8.16 ± 2.75 and for Population Border: 8.9 ± 8.3. The correlation of each cell's local density for example, was already observable by the human eye and therefore had to be normalized in order to perform hit-calling.

The technical features average to 6.1 ± 1.9 (Row Effects), 4.4 ± 2.1 (Column Effects) and 4.5 ± 2.71 (Spot Border). Analysis has been done on the individual plate level and thus, for the feature addressing the plate effect, no binning has been performed. We calculated AUC values for the positive and negative controls given in the screen after normalizing against the population context and against the technical features, and applied our approach for statistical hit scoring as presented in this study. We received increased performance (AUC = 0.66) in comparison to an analysis without normalizing against the features (AUC = 0.58).

## Conclusions

We have introduced a novel approach for the statistical processing of high-content cellular screens. We have shown that the population context of individual cells influences viral infection efficiency, and we have presented a normalization procedure to remove these effects in microscopy-based screens. We have developed a statistical testing procedure that takes into account individual cell measurements in hit-scoring, and we have demonstrated significantly improved sensitivity and specificity using this approach on two large-scale RNAi infection screens.

An evaluation that is based on individual cell measurements can exploit the information contained in hundreds of cells and thereby addresses the biological variability of cells, for example, cells that are in different states of the cell cycle. Obviously, the cells within one spot are treated in the same way and are not technical replicates as they are not independent of each other. Nevertheless, we identified two clearly separated distributions of cells within one spot which shows that there are phenotypic differences between individual cells even if they are treated in the same way. Our results show that the integration of multidimensional phenotypes from high-content screens can make data analysis much more sensitive and specific. Taking the individual cell measurements within each spot into account and using the computed p-values for individual spots highly improves sensitivity and specificity values, where the number of false positives and false negatives on a single spot level is limited to a minimum resulting in an almost perfect classification.

An interesting observation from previous infection screens is that there is a very low overlap of identified hits between different screens targeting the same virus [32]. While the situation is somewhat improved when considering overlaps at the pathway level, there is still a surprisingly high variability in results. Based on the significant influence of cell population context shown in both our screens as well as previously reported by Snijder et al. [18], it is very likely that population factors contribute at least partially to the problem. Our comparative analysis using average signal intensities, the clustering approach based on Ripley's k function and the CELL-BASED analysis with correction for population effects shows significant differences in resulting hit lists on the same screen, strongly advocating for normalization procedures accounting for cell population context. Looking at the enriched pathways identified with the hit lists using the AVERAGE and CELL-BASED approach, our results clearly show an increased sensitivity. We found substantially more pathways than when using the AVERAGE (with RIPLEY no pathways could be identified) where several of them have already been shown to be related in the processes of HCV and some are newly discovered.

Interestingly, Snijder et al. [18] showed that the location of a cell at the edge or in the middle of a local cell cluster is the main factor influencing dengue virus infection. In contrast, our results indicate that the size of the cell is the most significant population factor determining infection efficiency - both for dengue and hepatitis C virus. This difference may be due to the different platforms used, and the with associated differences in cell numbers and local cell density in each spot or well. Also, cell-line specific effects may contribute to these differences. Results reported by Snijder et al. were acquired in HeLa cells using 96 well plates with associated large wells and high cell numbers, whereas we used Huh7.5 cells on LabTeks, each containing 384 spots and without separating walls between different spots. It should further be noted that results presented by Snijder et al. did not include any effects of transfection reagents and siRNA knockdown, which may further alter the behavior of the cells.

We re-analyzed a previously published hepatitis C virus screen, and provided a comparative analysis of results using three different approaches. Our results show significantly improved sensitivity over previous data analyses on a single hit, as well as on the pathway level, which we attribute to both the removal of confounding population effects, as well as the exploitation of data from individual cells in hit scoring and statistical testing. Using the approach presented here, we could show high overlap of host pathways involved in hepatitis C virus and dengue virus infection, underlining the close evolutionary relationship between these two viruses. Our results on a non-viral screen strongly indicate that the population context not only influences infection RNAi screens, but generally applies. However, virus screens are more complicated as the infection itself can induce viral phenotypic effects (cytopathic effects) which may directly influence a population context feature. An infection, for example, may lead to larger cell sizes of the infected cells and normalizing against the cell size would destroy effects of perturbations. Since in the HCV and DENV there are no control spots without infections, we cannot test whether the cells in our screens suffer from cytopathic effects. However, the analysis with GSEA-ONLY, where we do not normalize against cell population effects, results in similar sensitivity and specificity values on controls, with the CELL-BASED method being even slightly better. This in combination with the increased sensitivity of the CELL-BASED analysis on the pathway level, indicates that we do not destroy effects when normalizing against cell context features, but allow for a more sophisticated and improved type of analysis. To conclude, high-content screening offers a powerful tool to further elucidate virus-host interactions in the future, with significant advantages over high-throughput screens with low-dimensional, non-microscopy based readouts. However, great care must be exercised in analyzing and integrating data, to fully exploit the potential offered by this platform.

## Methods

### RNAi screening

RNAi screens for host factors involved in HCV and DENV were carried out as previously described [17] on cell arrays (LabTek chambered coverglass slides). Seven different plates, each repeated twelve times (HCV), respectively six times (DENV), were used to knockdown a total of 719 different human kinase genes. Each kinase was individually targeted by three different siRNAs, using the Ambion *Silencer*^® ^Human Kinase siRNA Library V3 (AM80010V3). Reverse transfection of siRNAs into Huh7.5 cells was carried out as described by Erfle et al. [20]. After seeding of Huh7.5 cells, LabTeks were incubated for 36 hours (HCV) or 48 hours (DENV). For HCV, cells were infected with an HCV GFP reporter virus and 36 hours later immunostained with a GFP-specific antibody. For DENV, cells were infected with wild type Dengue virus (New Guinea C strain) and immunostained for viral envelope protein after 48 hours. Cell arrays were imaged with a scanning microscope (Scan^R, Olympus Biosystems) using a 10× objective (Olympus, cat. no. UPSLAPO 10×).

### Image Analysis and Quality Assessment

To analyze the image data of the siRNA screen, we developed an automatic image analysis system. The input of this system consists of two-channel images of each spot in the screen, corresponding to DAPI and GFP signals. In the DAPI channel, single cell nuclei were segmented using an edge-based approach. First, a binary image *f *was obtained from the input image *g *by combining the results of the gradient magnitude and the Laplacian operator:

$$f\left(x,y\right)=\left\{\begin{array}{c} 1\text{ if}|\nabla g|>T\text{ and}|\nabla^{2}g|<0\\ 0\text{ otherwise}, \end{array}\right)$$

where ∇ denotes the Nabla operator, $\left|\nabla g\right|=\sqrt{g_{x}^{2}+g_{y}^{2}}$, with $\nabla^{2}g=g_{xx}+g_{yy}$, with *g_x_*, *g_y _g_xx_*, *g_yy _*denoting first and second order partial derivatives of *g*, and *T *is a threshold which is automatically determined using the unimodal background symmetry method. The partial derivatives of g are determined using first and second order Gaussian derivative filters. Second, the whole connected components of pixels with the negative Laplacian which contained at least one pixel of the image *f *were selected. The majority of these pixels correspond to cell nuclei. Third, small connected components were removed and morphological closing as well as hole filling were applied to each single component. Finally, cell nuclei were identified from the connected components (segmented objects) by applying size, intensity, and circularity criteria, as previously described [33].

In the GFP channel, the viral protein production level (virus signal) of each cell was computed by the mean pixel intensity inside the nucleus neighborhood. We used non-overlapping rings around segmented nuclei as neighborhoods.

Quality filtering was performed eliminating out-of-focus images and image artifacts. On the single image level, images were automatically classified as low quality if they contained too large or too small a number of cell nuclei, or if the images were out-of-focus. The out-of-focus images were detected by measuring the gradient magnitude near boundaries of segmented cell nuclei. On the whole plate level, image quality and the result of automatic quality filtering were checked by a human, based on a whole plate view composed of image thumbnails overlaid with the automatic quality assessment results. In this view, for example, improperly stained plates were well visible and excluded from further analysis. To exclude apoptotic knockdowns or images with overlapping cells, spots with less than 125 or more than 500 cells were furthermore excluded from the analysis. Quality assessment resulted in an overall exclusion of 15% of the images due to quality problems.

### AVERAGE and RIPLEY data analysis method

AVERAGE: Data analysis on mean GFP intensities and cell counts were done in the statistical environment R http://www.r-project.org, using the cellHTS [10] and RNAither [11] packages from Bioconductor http://www.bioconductor.org. Loess normalization was used to decorrelate the GFP and DAPI channels, and the B-score method was used to remove spatial effects within plates and normalize for between-plate variability. The twelve (HCV), respectively six (DENV), replicate measurements were then summarized by the mean, and hits identified using a threshold of ± 1.5 and a p-value threshold of 0.05, using a t-test on the null-hypothesis of nonzero normalized GFP signal intensity per well.

RIPLEY: Data analysis using the Ripley's k-function based a clustering approach were performed on the raw images, as previously described [19].

### Calculation of cell context features and normalization using multiparametric regression

We computed five different cell context features and four technical features for each cell in the entire screen. (1) Size of each cell nucleus was directly computed from the nuclei segmentation in the DAPI channel. (2) The number of cells in each spot was counted based on the DAPI segmentation. (3) The location of cells within a local population (center or edge of a local cell population) was estimated by splitting each spot into a 15 × 15 grid. Then we counted the number of cells in each grid position. Grid size was manually optimized. Cells in a position adjacent to an empty field were identified as cells at the border of a local population. (4) Local cell density was estimated using a Gaussian kernel density estimator based on nuclei centers. (5) The shape of each cell was computed as the inverse of the cell's nucleus' circularity. We furthermore calculated four technical features for each cell: (1) Location of a cell in a spot (within spot or at the border), based on nuclei coordinates. (2) and (3): Row and column effects of the cell location, using the median signal intensity of all cells in the corresponding row or column of the LabTek as an estimator. (4) Overall plate effects on cell intensity were estimated using the median signal intensity of all cells on the corresponding LabTek. We then used multivariate adaptive regression splines as implemented in the "earth" package from CRAN http://cran.r-project.org to estimate residuals accounting for population and technical artifacts, on which further analysis was carried out.

### Estimation of within-bin and between-bin variability

To reduce dataset size for the estimation of effects on viral infection of the computed features, we used a binning procedure. Using 5% quantile steps on each feature 20 bins were computed. Only two bins were used for the binary features location of a cell with respect to local cell population and with respect to the spot. We then computed the average within-bin variability using the mean of the standard deviation of GFP signals for all cells assigned to a given bin, and the between-bin variability using the standard deviation of the mean GFP signals of all cells in each bin. To assess the effect of different bin sizes, we repeated the binning procedure based on different quantiles. We observed no significant differences in within- and between-bin variability. The within-bin and between-bin variability ratio was then calculated for each feature by dividing the between bin standard deviation by the average within bin standard deviation

### Individual cell-based hit identification

Residual GFP values of individual cells, after correction for cell population and technical features as described above, were used to identify host dependency factors. Data analysis was carried out by borrowing ideas from gene set enrichment analysis (GSEA). GSEA is one of the most popular strategies for detecting differentially expressed gene sets, and was first introduced in the field of gene expression analysis by Mootha et al. [23] and Subramanian et al. [34]. The method is essentially a weighted Kolmogorov-Smirnov test [24], which is applied to a running sum statistic over ranked gene lists, counting how often genes are or are not in the gene set of interest. The variant used here has been proposed by Sweet Cordero et al. [22], and corresponds to the standard, unweighted Kolmogorov-Smirnov test applied on two running sums which denote the number of sorted differentially expressed genes which are or are not in the given gene set. Using GSEA in the context of RNAi screens required basically one change of the original usage. Unlike in the analysis of gene expression data, the sets were defined not by genes but by cells coming from one spot, one siRNA or one gene.

For GSEA we started with a list *D *with *N *samples and computed a statistical score based on the correlation of the measurements (*g_j_*) to the phenotype of interest for all *j *= 1, ..., *N*. A variety of statistical scores, e.g., the t-score or signal-to-noise-ratio can be used in this step [31]. Based on this score, the list *D *was sorted and a running sum statistic *RS *was calculated for each predefined collection of genes *G*_1_, *G*_2_, ..., *G_m_*. The sorted list was processed from top to bottom and two running sums $R{S_{G}}_{{}_{k}}$ and $RS_{{Ḡ}_{k}}$ were computed. $R{S_{G}}_{{}_{k}}$ was increased by one, each time a sample belongs to *G_k _*and $RS_{{Ḡ}_{k}}$ each time a sample belongs to the complentary set ${Ḡ}_{k}$:

(1) $$RS_{G_{k}\left(i\right)}=\underset{\underset{j\leq i}{g_{j}\in G_{k}}}{\sum }\frac{1}{N_{G_{k}}}$$

(2) $$RS_{{Ḡ}_{k}\left(i\right)}=\underset{\underset{j\leq i}{g_{j}\notin G_{k}}}{\sum }\frac{1}{N-N_{G_{k}}}$$

where $N_{G_{k}}$is the number of *g_j _*∈ *G_k_*.

Finally, an enrichment score $ES_{G_{k}}$ for each *G_k _*was defined as the maximal deviation from zero of the difference of the running sum *G_k _*and its complementary set ${Ḡ}_{k}$:

(3) $$DIF_{i}\left(G_{k},{Ḡ}_{k}\right)=RS_{G_{k}}\left(i\right)-RS_{{Ḡ}_{k}}\left(i\right)$$

(4) $$ES_{G_{k}}=DIF_{j}\left(G_{k},{Ḡ}_{k}\right),\text{where }j=\arg\underset{i}{\max}|DIF_{i}\left(G_{k},{Ḡ}_{k}\right)|$$

In the case of RNAi experiments, cells within each single spot *k *were considered as the predefined sets *G_k _*and then the accumulation on top or bottom of the sorted list of all cell intensities in the screen was evaluated. Computation of the running sums was done by ranking the normalized GFP intensities in increasing order. The sign of the enrichment score determined the direction of the effect observed, i.e. positive *ES *corresponds to siRNAs having a negative effect on the phenotype (dependency factors).

To assess the significance of the obtained *ES *we used permutation testing. We permuted the cells of each plate and calculated an *ES *on the permutations (*ESperm*) for each spot based on the cells which have been assigned with the corresponding spot during the permutation. Then, using the median of all replicate plates *ESperm *was summarized using median and the resulting distribution was used to calculate the significance levels for the *ES *of the observed, unpermuted data. The bonferroni method was then used to account for multiple testing.

## Authors' contributions

IR, AK, MB and HE performed RNAi screens and image acquisition, HE and RB developed and conceived the experiments and advised on experimental work. PM and KR performed image processing. BK, RE, NAK and LK designed the statistical methods. BK and LK implemented the methods, performed the data analysis and wrote the manuscript. All authors have read and approved the final manuscript.

## Supplementary Material

Additional file 1 **Software**. (Archive containing R source code, example dataset and documentation in PDF-Format) This archive contains the R source code used for computation of the features, normalization and hit-calling, as well as an example data file (ASCII text) and a brief documentation in PDF-Format. Click here for file

Additional file 2 **Population context features**. Mean and standard deviation of the two population context features Cell Density and Cell Shape of the two screens in the twenty individual bins. Click here for file

Additional file 3 **Gene hit lists identified for HCV using the different methods analyzed in this work**. Gene hit lists of HCV screen after CELL-BASED, AVERAGE and RIPLEY analysis, as well as MARS-ONLY and GSEA-ONLY. Shown are genes that scored as hits using at least one of these methods. Enrichment scores are shown for CELL-BASED and GSEA-ONLY, z-scores for MARS-ONLY and AVERAGE and clustering scores for RIPLEY. Non-significant values are represented by empty cells. The last column indicates whether the respective gene also came out as a hit in the DENV screen, shown are enrichment scores from the DENV screen. Again, non-significant scores are left blank in the table. Click here for file

Additional file 4 **Gene hit lists identified for DENV using the CELL-BASED method**. Gene hit list of the DENV screen, using the CELL-BASED method. Shown are enrichment scores after normalization for cell population context and technical artifacts in the screen. Click here for file

Additional file 5 **Enriched pathways for HCV using DAVID**. Results of geneset enrichment analysis for KEGG and Biocarta pathways, using the DAVID bioinformatics software. Significant signaling pathways of the HCV screens are shown. The column "Category" shows whether the hit was found in the KEGG or BIOCARTA database. "Term" describes the name of the pathway. "Count" and "%" indicate how many genes and what percentage of the hit list are in the respective pathway. The column "PValue" shows the significance of the enrichment. Respective hit genes are listed in column "Genes". "List" is the total number of genes in the respective pathway. "Fold Enrichment" expresses the enrichment score and "Bonferroni", "Benjamini" and "FDR" show the p-value after the repective correction for multiple testing. Enriched pathways using the AVERAGE screen are shown additionally in S3. Click here for file

Additional file 6 **Enriched pathways for DENV using DAVID**. Results of geneset enrichment analysis for KEGG and Biocarta pathways, using the DAVID bioinformatics software. Significant signaling pathways of the DENV screens are shown. The column "Category" shows whether the hit was found in the KEGG or BIOCARTA database. "Term" describes the name of the pathway. "Count" and "%" indicate how many genes and what percentage of the hit list are in the respective pathway. The column "PValue" shows the significance of the enrichment. Respective hit genes are listed in column "Genes". "List" is the total number of genes in the respective pathway. "Fold Enrichment" expresses the enrichment score and "Bonferroni", "Benjamini" and "FDR" show the p-value after the repective correction for multiple testing. Click here for file
