# Supplementary material for: Normalizing for individual cell population context in the analysis of high-content cellular screens
Source: BMC Bioinformatics. 2011 Dec 20;12:485. doi: 10.1186/1471-2105-12-485 (PMC3259109; doi:10.1186/1471-2105-12-485)
Supplement: Additional file 1 — Software. (Archive containing R source code, example dataset and documentation in PDF-Format) This archive contains the R source code used for computation of the features, normalization and hit-calling, as well as an example data file (ASCII text) and a brief documentation in PDF-Format. [file 1471-2105-12-485-S1.GZ › documentation.pdf]

Normalization for Population Context in High-Content Screens

Bettina Knapp, Ilka Rebhan, Anil Kumar, Petr Matula, Narsis A. Kiani, Marco Binder, Holger Erfle, Karl Rohr, Roland Eils, Ralf Bartenschlager and Lars Kaderali

Abstract

Motivation:

High-content, high-throughput RNA interference offers unprecedented possibilities to elucidate gene function and involvement in biological processes. Microscopy based screening allows phenotypic observations at the level of individual cells. It was recently shown that a cell's population context significantly influences results. However, standard analysis methods for cellular screens do not currently take individual cell data into account; the potential of high-content screening is thus not fully tapped.

Results:

We present a method to normalize and statistically score microscopy based RNAi screens, exploiting individual cell information of hundreds of cells per knockdown. Each cell's individual population context is employed in normalization. We show results on two infection screens for hepatitis C and dengue virus, both showing considerable effects of population context on infection. Using cell-based analysis and normalizing for population context, we achieve significantly improved sensitivity and specificity, leading to the identification of new host factors and higher reproducibility of results.

Software

Software has been implemented in R (http://www.r-project.org).

Software License

This program is free software: you can redistribute it and/or modify it under the terms of the GNU General Public License as published by the Free Software Foundation, either version 3 of the License, or (at your option) any later version.

This program is distributed in the hope that it will be useful, but WITHOUT ANY WARRANTY; without even the implied warranty of MERCHANTABILITY or FITNESS FOR A PARTICULAR PURPOSE. See the GNU General Public License for more details.

Files

By using the software, you agree to the terms of the GNU General Public License.

The following files are contained in the archive:

File Description	File Name
R-code for population feature computations	density.R
R-code for normalization and hit scoring	normalize.R
Example dataset	data.txt
This file	documentation.pdf

Example Data Analysis

Image Processing

Image processing can be carried out using commercial software such as ScanR, or publicly available software such as EBImage. Our results are based on the image processing pipeline published in Single-cell-based image analysis of high-throughput cell array screens for quantification of viral infection, Matula et al., Cytometry A, Volume 75A, Issue 4, pages 309-318, April 2009.

Input data format:

Raw input data for each plate must be provided in a tab-delimited text file, containing the following columns, and with each row corresponding to data from one cell in the assay:

Column Name	Explanation	Example
**WellNo**	Number of Well	1
**CellId**	A numeric identifier for the cell	312
**NucleiSize**	Size of cell nucleus	625
**NucleiP2A**	1/Circularity of Nucleus	1.23954
**NucleiXpos**	X-Position of Cell	870.747
**NucleiYpos**	Y-Position of Cell	890.598
**CytoplasmSize**	Size of Cell Cytoplasm	477
**CytoplasmMean**	Mean signal intensity in Cytoplasm	703.447
**CytoplasmStdDev**	Std. deviation of signal intensity in Cytoplasm	419.354
**CytoplasmInsideNucleusMean**	Mean signal intensity of Nucleus	444.608
**CytoplasmInsideNucleusStdDev**	Std. deviation of signal intensity in Nucleus	231.289
**CytoplasmOverexposedCount**	Number of saturated pixels (currently unused)	0
**CytoplasmBgCorrectedMean**	Background corrected mean cytoplasm signal	494.665
**CytoplasmBgCorrectedStdDev**	Standard deviation of bg-corrected Cytoplasm signal	418.334
**PositionClass**	1 = Cell is within spot, 0 = Cell is outside of spot	1
**CellClass**	1 = Object recognized as cell; 0 = Object is no cell and will be ignored	1
**ExpressionClass**	1 = Cell has associated signal, 0 = cell has no	1

	signal, will be ignored	

Computation of population context from data

The following R-commands read in the raw data and carry out the computations of the population context parameters. Start R, and then type:

```
source("density.R") # load the R-function required
runme("data.txt","data_processed.txt") # carry out computations
```

These computations require considerable amounts of memory, and will take some time. This must be done for each of the input files, and will produce an output file containing the input data plus computed population features.

Results will be stored in a file data_processed.txt. This file is the basis for further data analysis.

Data normalization and hit scoring:

The next step then is to normalize this data, and carry out the hit scoring:

```
source("normalize.R")

# plate layout
gridsize <- c(32,12) # size of plate is 32 by 12 spots
well_no <- 384 # number of spots is 384
ctrl.pos <- c(81,86) # locations of positive controls (spot number)
ctrl.neg <- c(8,72) # locations of negative controls (spot number)
ctrl <- c(ctrl.pos,ctrl.neg)
labtekno <- 1 # number of plates - only 1 in this example
replicateno <- 1 # number of replicates - only 1 in this example
annot <- paste("gene",seq(1,well_no)) # gene names - here just numbered
consecutively

# Run the analysis routine:
results <-
score("data_processed.txt",gridsize,well_no,ctrl,labtekno,replicateno,annot
)
```

The result of these computations is a list containing the p-values, variance ratios for all features, and normalized data.
